# Explaining Children’s News Avoidance During the COVID-19 Pandemic

**DOI:** 10.3389/fpsyg.2022.889096

**Published:** 2022-06-22

**Authors:** Ming Ebbinkhuijsen, Moniek Buijzen, Rebecca de Leeuw, Mariska Kleemans

**Affiliations:** ^1^Behavioural Science Institute, Radboud University, Nijmegen, Netherlands; ^2^School of Social and Behavioural Sciences, Erasmus University Rotterdam, Rotterdam, Netherlands

**Keywords:** news avoidance, news consumption, parental mediation, reactive coping, emotional responses, COVID-19 pandemic

## Abstract

Despite growing concerns that children (8–13 years old) tend to avoid the news, the reasons why have received little research attention. Therefore, the current study aims to develop and test a model conceptualizing the relations between children’s news consumption, news avoidance, emotional responses (negative emotions and anxiety-related behaviors), and parent and child mitigation strategies. The model was tested using data collected during the first year of the COVID-19 pandemic. The current, preregistered, survey study was part of a longitudinal project and used data from the second wave. Data were collected in November/December 2020 among 510 children (*M_*age*_* = 10.40; 53.72% girls). Findings showed that children who consumed more news during the pandemic avoided pandemic news less often. Children who experienced more anxiety-related behaviors regarding pandemic news avoided pandemic news more often. The relation between news consumption and emotional responses was stronger for children who experienced restrictive parental mediation more often, indicating that this was not an effective parental mediation strategy for tempering their emotional responses. Children with higher levels of emotional responses used reactive coping strategies more often. However, this did not seem to be an effective strategy against pandemic news avoidance because none of the strategies had a negative relation with pandemic news avoidance. Distancing was even positively related to pandemic news avoidance. Although the current study was not able to fully unravel how news avoidance-related constructs relate to one another, we were able to get some important insights guiding future research. Specifically, it is of crucial importance to unravel the mechanisms that increase the chance of children’s news avoidance and those that mitigate it, to build interventions to counteract news avoidance and to protect children from the negative emotional consequences by news consumption.

## Introduction

In the beginning of the COVID-19 pandemic, people tended to consume more news and used a bigger variety of news sources to be informed about the pandemic (Noel, 2020; Common Sense Media, 2021; De Bruin et al., 2021). However, in the following months people increasingly indicated to avoid the news in general and pandemic news specifically (Fletcher et al., 2020; De Bruin et al., 2021). According to De Bruin et al. (2021), this decrease in news consumption was caused by information overload and the experience of negative emotions during news consumption. These studies contribute to growing attention in scientific research for the question why people avoid the news. Surprisingly, younger age groups are hardly investigated in this regard. Research on news consumption of youth conducted before the pandemic showed that half of them have low overall news use and can therefore be conceptualized as news avoiders. Moreover, compared to more frequent news consumers, participatory engagement is lower for these news avoiders (Edgerly et al., 2018).

The current study focuses on children as they are in their formative years as news consumers. When children are about 8 years old, they reach the age at which they become able to distinguish fiction from reality, develop an increasing interest in what is going on in the world, and want to be taken seriously and seen as (future) citizens in society (Kandemir-Ozdinc and Erdur-Baker, 2013; Alon-Tirosh and Lemish, 2014; Valkenburg and Piotrowski, 2017). In other words, news consumption becomes interesting and relevant for them during this life phase. At the same time, concerns are raised that news consumption might trigger news avoidance in this age group specifically, because of the negative nature that news stories generally have and the negative emotional responses it consequently elicits in children (Riddle et al., 2012; Alon-Tirosh and Lemish, 2014; Ebbinkhuijsen et al., 2021a,b).

To shed more light on this, the present study takes the first step in disentangling the mechanisms related to children’s news consumption and news avoidance. The COVID-19 pandemic provides a highly suitable context to test our model of news avoidance, representing a time with particularly impactful and potential frightening news for children (Phelps and Sperry, 2020).

## A Model of News Avoidance

At the core of a model explaining news avoidance lies the relation between children’s news consumption, subsequent emotional responses (i.e., negative emotions, anxiety-related behaviors), and their news avoidance. Additionally, it includes parent and child mitigation strategies. Specifically, the model maps how parental mediation and reactive coping strategies interact with this core relation and thus may affect children’s news avoidance.

### Main Relation: News Consumption and News Avoidance

Skovsgaard and Andersen, (2020, p. 463) define news avoidance as “low news consumption over a continuous period of time caused either by a dislike for news (intentional) or a higher preference for other content (unintentional).” Intentional news avoidance causes the most concerns because people explicitly decide not to follow the news (anymore). Based on past research, Skovsgaard and Andersen (2020) discerned three reasons for news avoidance: (1) the news is too negative, (2) the news cannot be trusted, and (3) there is too much news.

All three reasons may explain why news concerning the COVID-19 pandemic increased news avoidance. Regarding the first, the negative focus of news in general has shown to negatively affect news consumers and their well-being over time (Skovsgaard and Søberg, 2016; Boukes and Vliegenthart, 2017, in Skovsgaard and Andersen, 2020). In addition, in-depth interviews with participants from five different countries showed that especially young adults (18–30 years old) avoid the news because tragedies in the news affect them emotionally (Villi et al., 2022). Concerning the COVID-19 pandemic, news was often negative, influencing one’s emotional state as well (De Bruin et al., 2021). In April 2020, two-third of people felt the need to take breaks from COVID-19 news (Dobson-Lohman and Potcovaru, 2020; Sheares et al., 2020).

Related to the assumption that a lack in trust may cause news avoidance, past research showed that people perceive the media as biased and therefore do not know what to believe (Newman and Fletcher, 2017; Toff and Nielsen, 2018). The spread of fake news and misinformation makes it harder to distinguish what to believe and what to trust (Xiao et al., 2021). Especially during the COVID-19 pandemic, there was a serious spread of fake news and of news containing misinformation about the pandemic – which both could cause a mistrust in news (Roozenbeek et al., 2020; Zarocostas, 2020). 72% of adults indicated that they do not know what is true and false about COVID-19 and 77-79% of them indicated to avoided news because they have encountered a lot of misinformation (Dobson-Lohman and Potcovaru, 2020; Sheares et al., 2020).

Finally, the abundance of news may cause news avoidance during the COVID-19 pandemic. In general, news narratives demand cognitive skills from news consumers as they have to process this new information (Lăzăroiu et al., 2017). Especially the rapid sharing of information puts news consumers under pressure and may overload their cognitive abilities (Gunter, 2015; Lăzăroiu et al., 2017). These feelings of news overload could lead to news avoidance as well (Song et al., 2017). According to the in-depth interviews of Villi et al. (2022), feeling overloaded or fatigued by the news was indeed discerned as one of the reasons why people avoid the news situationally or topic-specifically (e.g., feeling fatigued by Trump-news) or structurally. These cognitive drivers might have played a role during the pandemic as well. That is, a huge amount of information was available, which was even described as an infodemic (Zarocostas, 2020). 35–46% of adults reported feeling overwhelmed by the amount of COVID-19 news (Dobson-Lohman and Potcovaru, 2020).

Although there is thus reason to expect an increase in news avoidance during the pandemic, it was found that news consumption initially increased among adults (Kalogeropoulos et al., 2020; De Bruin et al., 2021) and youth (Noel, 2020; Common Sense Media, 2021). People consulted television and radio as source of information about the pandemic most often, followed by officials and social media (Lăzăroiu and Adams, 2020). Among children this also appears to be the case as, for example, viewer ratings of the Dutch children’s television news program “NOS Jeugdjournaal” [NOS Youth News] increased enormously in March 2020 (Mediacourant, 2020). In other countries, such as the United Kingdom, Spain, and the United States, a similar pattern of increased news consumption among children in the first months of the COVID-19 pandemic was found (Nielsen, 2020; Ofcom, 2020; Masip et al., 2021).

In-depth interviews with Belgian news users (21–33 years old) who experienced changed news habits since the beginning of the pandemic provide insights into the contradictory explanations of increased news consumption and news avoidance (Vandenplas et al., 2021). Initially, people wanted to be up to date about the measures and numbers, wanted to share their insecurities, talked about the news with others, and believed that the news about COVID-19 was inescapable. After a while, people felt overloaded with news covering COVID-19 related topics, and experienced negative effects on their well-being due to the negative tone and content of the news. This could be the case for children as well.

In line with Skovsgaard and Andersen (2020), it is expected that children who consumed more news during the pandemic, had a higher chance of seeing negative content. Moreover, they were more likely to be exposed to (discussions on) fake news and misinformation. And finally, the amount of COVID-19 news could easily have elicited feelings of overload^1^. Therefore, we expect^2^ that news consumption during the pandemic increases children’s news avoidance:

*H1:* The more children consume news during the COVID-19 pandemic, the more they avoid pandemic news.

### Mediation *via* Negative Emotions and Anxiety-Related Behaviors

Emotional responses elicited by children’s news consumption can be discerned into two categories; negative emotions – such as fear, worry and sadness – and anxiety-related behaviors – such as having nightmares, stomach aches and rumination (cf. Smith and Moyer-Gusé, 2006; Buijzen et al., 2007).

Several studies have shown that children display increased negative emotions after negative news consumption (e.g., Walma Van Der Molen et al., 2002; Buijzen et al., 2007; Riddle et al., 2012; Ebbinkhuijsen et al., 2021b). In general, the intensity of emotions elicited by the news was found to be relatively low in studies that investigated children’s consumption of news that is specifically tailored to their needs – such as the Dutch children’s television news (e.g., Kleemans et al., 2017a,b; Ebbinkhuijsen et al., 2021a). However, children can also easily access and be exposed to non-childproof news content in an online setting (cf. Apestaartjaren, 2020). This might elicit more intense negative feelings. In addition, experiencing anxiety-related behaviors in response to news – such as having nightmares, stomach aches and rumination (Smith and Moyer-Gusé, 2006) – is also worrisome and not beneficial for children.

For COVID-19 news in particular, parents reported that 54.7% of their children displayed a negative reaction, such as being frightened, disturbed, or upset (Cantor and Harrison, 2022). Parents also described their children’s reactions as feeling overwhelmed, terrified, confused, and freaked out. They indicated to receive lots of questions from their child(ren), reflecting feelings of insecurity and uncertainty about this novel situation for them (Cantor and Harrison, 2022).

Therefore, we model a 2-step indirect relation (mediation) between news consumption and news avoidance, with negative emotions and anxiety-related behaviors as explaining mechanisms:

*H2:* The more children consume news during the COVID-19 pandemic, the more they report experiencing (a) negative emotions and (b) anxiety-related behaviors regarding the pandemic.

*H3:* The more children report experiencing (a) negative emotions and (b) anxiety-related behaviors regarding the COVID-19 pandemic, the more they report to avoid pandemic news.

### Parental Mediation as Moderator Between News Consumption and Emotional Responses

As a final step in the model, we examine how parent and child mitigation strategies can temper emotional responses to news to reduce or counteract children’s news avoidance.

Parents try to regulate children’s television (news) viewing by applying parental mediation strategies (Valkenburg et al., 1999, 2013; Nathanson, 2001). The literature distinguishes three strategies: active mediation, restrictive mediation, and coviewing. *Active mediation* involves discussions of television content between parent and child to help understand what they see on television (news) and what it really means (Valkenburg et al., 1999, 2013). These discussions might entail negative (e.g., disagreeing with the way the content is presented), positive (e.g., approving the content or being enthusiastic about it), and neutral comments (e.g., explaining the information that is presented) on the media content (Nathanson and Eveland, 2019). *Restrictive mediation* includes parents deciding what their child can and cannot watch when it comes to specific programs or news outlets (Valkenburg et al., 1999, 2013). According to Nathanson and Eveland (2019), parents apply restrictive mediation more often for younger children and when they are concerned about the negative impact of exposure to (news) media on their children. Nathanson and Eveland (2019) also argue that restrictive mediation has more to do with general family rules about media content and less to do with specific news content. *Coviewing* means that parent and child watch television (news) together because they have a common interest in the content or both like the program (Valkenburg et al., 1999, 2013; Nathanson and Eveland, 2019).

Although it is assumed that parental mediation is helpful in counteracting negative influences of television consumption, previous research has shown that not all strategies are as effective when it comes to children’s news consumption and negative emotions. For example, in a study on the effects of children’s exposure to a violent news event on negative emotions, active mediation decreased feelings of fear, worry, anger, and sadness in children after exposure to the negative event. However, children from parents who applied the restrictive mediation strategy reported higher levels of fear and worry concerning this news (Buijzen et al., 2007). This suggests that restrictive mediation can have no or even an opposite effect when it comes to parental interference in children’s news consumption.

In line with this, a recent study on parental mediation of COVID-19 news in children (6-13 years old), showed that active mediation was related to lower lability/negativity in children – which was measured as the presence of excessive exuberance, disruptive behaviors, and anger. Coviewing was not related to higher or lower levels of lability/negativity, and restrictive mediation was related to higher lability/negativity in children (Morelli et al., 2022). Following the results from past research, we expect:

*H4:* The relation between news consumption during the COVID-19 pandemic and (a) negative emotions and (b) anxiety-related behaviors regarding the pandemic is moderated by parental mediation, with a stronger relation for restrictive parental mediation than for active parental mediation and coviewing.

### Reactive Coping as Mediator Between Emotional Responses and News Avoidance

Reactive coping strategies are ways to deal with stressful situations that happen at this moment or happened in the past (Schwarzer and Taubert, 2002). News events can be an example of a stressful situation because the external demands appraised by news might be higher than the cognitive and emotional abilities of children in dealing with it (cf. Lazarus and Folkman, 1984, p. 131). According to Causey and Dubow (1992) five general coping strategies for children can be discerned: seeking social support from peers or adults, problem solving, distancing, internalizing (“responses that are turned inward”, Causey and Dubow, 1992, p. 49), and externalizing (“responses that are turned outward”, Causey and Dubow, 1992, p. 49). In the context of news, children cannot have an influence on the presented news event (e.g., change the event), thus problem-solving strategies seem to be irrelevant here. The other coping strategies might serve as mediating factors between emotional responses and news avoidance.

To be more specific, past studies provide some indications that coping strategies are effective in dealing with the negative consequences of negative news exposure. For example, Hoffner and Haefner (1994) investigated news avoidance concerning exposure to news about the Gulf war. They found that children who were more enduring upset during the Gulf war, avoided exposure to news about the war more often. They argue that news avoidance might prevent children from experiencing more negative emotions and provide them the opportunity to use distraction as a coping strategy for protecting themselves from this news. Two other studies investigated whether seeking social support in peers would be an effective strategy to deal with (negative) emotions elicited by the news (Kleemans et al., 2017b; Ebbinkhuijsen et al., 2021b). Real-live conversations with peers seems to be a potentially successful coping strategy for these children (Kleemans et al., 2017b). However, Ebbinkhuijsen et al. (2021b) found that distancing – and more specifically distraction – is more effective than seeking online social support in peers when dealing with negative emotions elicited by the news, directly after exposure to negative news content.

A recent study on parent reports of children’s fright reactions to pandemic news showed that parents use several coping strategies to help their child (8–12 years old) to cope with this news, such as reducing their news consumption, answering questions they have, and distracting them with a different activity. Parents evaluated their attempts to use these strategies as somewhat effective to very effective (Cantor and Harrison, 2022).

Taken together, we expect reactive coping strategies to be effective mediators in the relation between emotional responses and news avoidance:

*H5:* The relation between (a) negative emotions, (b) anxiety-related behaviors regarding the COVID-19 pandemic and pandemic news avoidance is mediated by reactive coping strategies, with (H5a) negative emotions/anxiety-related behaviors regarding the pandemic resulting in more reactive coping, subsequently (H5b) leading to a decrease in pandemic news avoidance.

Although there are several studies that investigated children’s news consumption and the use of reactive coping strategies (Hoffner and Haefner, 1994; Kleemans et al., 2017b; Ebbinkhuijsen et al., 2021b; Cantor and Harrison, 2022), we were not able to make a specific prediction which coping strategies would be stronger in mediating the relation between (a) negative emotions, (b) anxiety-related behaviors regarding the COVID-19 pandemic and pandemic news avoidance. Therefore, we question:

*RQ1:* To what extent do the five reactive coping strategies (seeking social support in peers, seeking social support in adults, distancing, internalizing, externalizing) differ in mediating the relation between (a) negative emotions, (b) anxiety-related behaviors regarding the pandemic and pandemic news avoidance?

### Testing the Model

**FIGURE 1:**
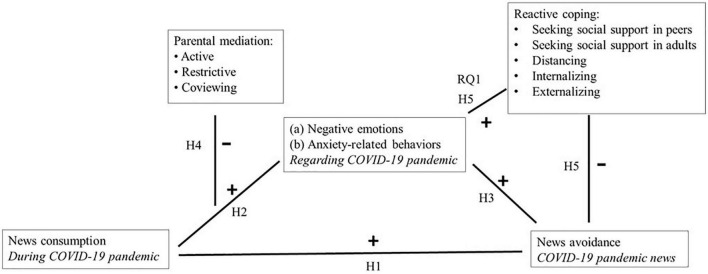
Conceptual model.

Figure 1 depicts the conceptual model based on the hypotheses and research question. The model is developed based on the theoretical and empirical insights described above and will be tested in the COVID-19 pandemic context.

## Methods

### Design

The study was part of a longitudinal project conducted in the Netherlands, in which children received an online survey every two months between June 2020 and May 2021. To test our model explaining children’s news avoidance, we used the data of the questionnaire in Wave 2 (November/December 2020). The study was preregistered^3^ and received the approval of the ethics committee of the host university (ECSW-2020-086). The data and syntax of our analyses (R-script) are available open access at the DANS Easy data repository.^4^

### Procedure and Participants

Participants for the longitudinal project were recruited using an information letter, which was distributed by schools to parents of children in grades 4, 5, and 6. We first contacted schools to ask whether they were willing to distribute the information letter. In this letter, parents were informed about the study and were asked to give active consent for participation of their child through a link (Qualtrics survey). After parents provided consent, they received an email with a link to the first questionnaire for their child. In this questionnaire, we asked children if they wanted to participate in this study (assent). If they agreed, they could continue with the questionnaire. Children were told they could win a gift card by participating, raffled under the participants at different measurement points. As a surprise, they all received a small present at the end of the project.

Originally, we planned to recruit all participants in June 2020. However, because primary schools had just re-opened again after being closed due to the pandemic and because the summer holiday was approaching, it was hard to find schools willing to distribute our information letter and to reach enough participants. We thus decided to recruit additional participants in September 2020. Diverging from our preregistration, we decided to test the model for this study using the measurement of Wave 2 (November/December 2020) for all participating children. The main reason to do so was because we preferred to use responses from the same measurement period as time differences may distort the results. In November/December 2020, all participating children in the study answered the questions under comparable circumstances, giving a more solid basis for testing the model. Moreover, we noticed an inconsistency in the formulation of one of the variables we want to investigate. We aimed to develop a model in which we measure children’s negative emotions regarding COVID-19 news and their reactive coping strategies. However, coping was measured for the emotion “fear” only, which we changed in the measurement for Wave 2 to all “negative emotions” (see^5^; note for Study 1).

We aimed to recruit a panel of 1,000 participants for our longitudinal study, who attended grade 4, 5, or 6 of primary school when they first participated in this study. This sample size was based on earlier longitudinal studies using SEM, and the expected attrition (e.g., Fikkers et al., 2019; Geusens and Beullens, 2021). After recruiting participants in both June (referred to as premeasurement) and September 2020 (Wave 1), we received consent forms of *N* = 975 parents.

For the current study, we needed at least 500 participants (*d* = 0.20, power = 0.80, 12 latent and 3 observed variables, alpha = 0.05; Soper, 2021). In total, *N* = 554 children participated in the survey for Wave 2 (November/December 2020). However, in line with our preregistration, *n* = 44 responses had to be excluded from the analysis (cf. Meade and Craig, 2012). Children who filled out the same questionnaire twice (*n* = 4), responses that could not be connected to parents’ consent (*n* = 6), questionnaire there were completed less than half (*n* = 16), or careless responses – e.g., giving the same answer extremely often (*n* = 18; package careless in R; Yentes and Wilhelm, 2021) were removed. This resulted in a final sample of *N* = 510 children (53.72% girls; *M_*age*_* = 10.40, *SD*_*age*_ = 1.01, range 8–13 years old), who were attending grade 4 (*N* = 104), grade 5 (*N* = 201), grade 6 (*N* = 154). *N* = 45 children were in the first grade of secondary school, because they were in grade 6 before summer (premeasure).

### Measures

For all measures containing three or more items (except for news consumption), Kaiser-Meyer-Olkin measure of sampling adequacy, Bartlett’s test of sphericity, and Principal Factor Analyses (PFA) with oblique rotation (oblimin) based on the scree plot criterium were performed. As the three parental mediation strategies consisted of two items each, only reliability analyses for each mediation style individually were performed. For all final scales, mean scores were calculated (for reliability and descriptive statistics of all measures, see Table 1).

**TABLE 1:** Descriptive statistics for all measures.

Item	α	*M*	*SD*	*N*
News consumption	–	1.11	0.78	510
News avoidance	0.90	3.04	1.26	507
Negative emotions	0.88	2.22	0.83	510
Anxiety-related behaviors	0.75	1.38	0.59	507
**Reactive coping**				
Seeking social support in peers	0.80	2.61	1.28	495
Seeking social support in adults	0.87	3.19	1.40	494
Distancing	0.73	3.17	1.33	488
Externalizing	0.78	1.25	0.57	491
**Parental mediation**				
Active mediation	0.74	3.13	1.20	502
Restrictive mediation	0.60	1.99	0.99	502
Coviewing	0.71	2.86	1.18	501

*Package Psych was used for obtaining descriptive statistics (Revelle, 2021).*

#### News Consumption During the COVID-19 Pandemic (Independent Variable)

We asked children how often they consumed the Dutch children’s news program “NOS Jeugdjournaal” [NOS Youth News] in the past week with four items (on TV, *via* website or app, *via* Instagram, *via* YouTube) and how often they consumed other news in the past week (e.g., news for adults) with four items (on TV, *via* website or app, *via* Instagram, *via* YouTube), with response scales ranging from 0 = “*never*” to 7 = “*every day*.” Because these items did not require high internal reliability to measure the same construct (e.g., one can watch news very often *via* television, and never *via* a news website), we did not perform factor and reliability analyses for this variable. Item scores were averaged to create the news consumption variable.

#### COVID-19 Pandemic News Avoidance (Dependent Variable)

We used five statements for news avoidance (cf. Van den Bulck, 2006) and adjusted them to news about the pandemic. The statements were: “When the news about COVID-19 comes on, I switch to another channel,” “There is so much to follow in the media, that I seldom follow the news about COVID-19,” “Usually the news about COVID-19 isn’t interesting enough to follow it,” “If the news about COVID-19 annoys or bothers me, I change channels,” “Some days I really don’t want to follow the news about COVID-19.” Response options ranged from 1 = “*never*” to 6 = “*very often*.” The Kaiser-Meyer-Olkin measure of sampling adequacy, *KMO* = 0.88, and Bartlett’s test of sphericity, χ*^2^*(10) = 1483.83, *p* < 0.001, showed that performing a factor analysis was suitable for these 5 items. The PFA (oblimin) yielded a 1-factor solution that was best fit for these items. Factor loadings were between 0.83 and 0.87.

#### Negative Emotions Regarding Pandemic News (Mediator)

We asked children to indicate how much they experienced feelings of fear, worry, anger, and sadness caused by pandemic news with two statements for each emotion (cf. Buijzen et al., 2007). We also added two items about feelings of insecurity caused by pandemic news because at that time it was uncertain how the pandemic would develop. All items were measured on a 6-point scale ranging from 1 = “*never*” to 6 = “*very often*.” A PFA (oblimin) was conducted [*KMO* = 0.87; Bartlett’s test of sphericity, χ*^2^*(45) = 2317.87, *p* < 0.001], which resulted in two factors. The items measuring anger were removed as these seemed to measure a different construct based on high factor loadings on the second factor (0.89 and 0.95). A new PFA (oblimin) was conducted, which now resulted in one factor, with sufficient factor loadings (between 0.63 and 0.82).

#### Anxiety-Related Behaviors Regarding Pandemic News (Mediator)

Children indicated how often they suffered from negative behavioral reactions because of the news about the pandemic. We used five items from an existing scale (cf. Cantor et al., 1993; Cantor, 1996). These were: “difficulty sleeping,” “having nightmares,” “a desire to sleep with parent,” “difficulty eating,” “an upset stomach.” We added two items, because they were highly relevant for the situation at that moment: “staying as far away from other people as possible,” and “not daring to go to places with a lot of people.” All items were measured on a 6-point scale ranging from 1 = “*never*” to 6 = “*very often*.” *KMO* = 0.75 and Bartlett’s test of sphericity, χ*^2^*(21) = 753.14, *p* < 0.001, showed that performing a factor analysis was suitable. A PFA (oblimin) was conducted for these 7 items, which resulted in two factors. The last two items loaded on a separate factor, so we decided to remove these and conduct a new PFA (oblimin) with the original anxiety-related behaviors items (cf. Cantor et al., 1993; Cantor, 1996). Thereafter, items loaded on one factor with factor loadings between 0.67 and 0.80.

#### Parental Mediation (Moderator)

We measured three parental mediation strategies: active mediation, restrictive mediation, and coviewing (Valkenburg et al., 1999, 2013). These were measured on a 6-point scale ranging from 1 = “*never*” to 6 = “*very often*.” Questions were: “How often do your parents try to help you understand what happens in the news?,” “How often do your parents explain to you what the news really means?” (active mediation), “How often do your parents tell you not to follow shocking news?”, “How often do your parents specify where you are allowed to follow the news?” (restrictive mediation), “How often do you follow the news together with your parents because of a common interest in it?” and “How often do you follow the news together with your parents because you both like it?” (coviewing). For each parental mediation style, a reliability analysis was performed (see Table 1).

#### Reactive Coping (Mediator)

We asked children how often they did something when they experienced negative emotions by the news about the pandemic. We investigated five coping strategies: seeking social support in peers, seeking social support in adults, distancing, internalizing, and externalizing (cf. Causey and Dubow, 1992). Children answered statements – selected from Causey and Dubow (1992) – regarding these reactive coping strategies on a 6-point scale ranging from 1 = “*never*” to 6 = “*very often*”. For each coping strategy, separate PFAs were conducted.

##### Seeking Social Support in Peers

The statements presented to children were: “I tell a friend what I’ve heard,” “I talk to a friend about how it made me feel,” “I get help from a friend.” *KMO* = 0.69 and Bartlett’s test of sphericity, χ*^2^*(3) = 507.46, *p* < 0.001, showed that performing a factor analysis was suitable for these 3 items. A PFA (oblimin) showed a 1-factor solution. Factor loadings were between 0.83 and 0.89.

##### Seeking Social Support in Adults

Children answered these statements about seeking social support in adults: “I tell an adult about what I’ve heard,” “I talk to an adult about how it made me feel,” “I get help from an adult.” *KMO* = 0.72 and Bartlett’s test of sphericity, χ*^2^*(3) = 797.32, *p* < 0.001, showed that performing a factor analysis was suitable for these 3 items. A PFA (oblimin) yielded a 1-factor solution. Factor loadings were between 0.87 and 0.92.

##### Distancing

Statements presented to children for indicating how often they used distancing as a reactive coping strategy were: “I try to forget the whole thing,” “I tell myself it doesn’t matter,” “I do something to take my mind off of it.” *KMO* = 0.67 and Bartlett’s test of sphericity, χ*^2^*(3) = 319.68, *p* < 0.001, showed that performing a factor analysis was suitable for these 3 items. A PFA (oblimin) showed a 1-factor solution. Factor loadings were between 0.76 and 0.83.

##### Internalizing

The following statements were presented to measure internalizing: “I go off by myself,” “I worry too much about it,” “I cry about it.” *KMO* = 0.61 and Bartlett’s test of sphericity, χ*^2^*(3) = 108.81, *p* < 0.001, showed that performing a factor analysis was suitable for these 3 items. A PFA (oblimin) yielded a 1-factor solution. Factor loadings were between 0.66 and 0.75. However, the reliability of the scale was poor (α = 0.48). Removing items would also not have increased reliability, therefore this scale was not used for building the model and to test the hypotheses/RQ.

##### Externalizing

Externalizing was measured using the following statements: “I yell to let off steam,” “I curse out loud,” “I get mad and throw or hit something.” *KMO* = 0.69 and Bartlett’s test of sphericity, χ*^2^* (3) = 430.11, *p* < 0.001, showed that performing a factor analysis was suitable for these 3 items. A PFA (oblimin) yielded a 1-factor solution. Factor loadings were between 0.80 and 0.87.

#### Covariates

Participants’ grade, sex, and proximity of COVID-19^6^ were measured to be included as covariates. To assess proximity of COVID-19, we asked parents when filling out the informed consent form to what extent their child experienced the consequences of COVID-19 in their close proximity, measured on a 6-point scale ranging from 1 = “*not at all*” to 6 = “*a lot*” (*M* = 2.80, *SD* = 1.66, *N* = 498).

### Analysis Procedure

All analyses were performed using R (RStudio Team, 2019). First, descriptive statistics were obtained (see Table 1) and correlations between all variables were computed (see Appendix Table A1 in Appendix A). To develop a model for children’s news avoidance, we tested the relevant subsets of the model beforehand (see Appendix B). The subsets of the model had good fits; therefore, the complete model was tested. The model was tested using Structural Equation Modeling in R (Lavaan package; Rosseel, 2012). Full information maximum likelihood (FIML) was used as the imputation strategy and a robust estimator (MLR) was used. For assessing whether the model adequately fit the observed data, we looked at Goodness-of-Fit indices, which were χ*^2^*/df < 3, RMSEA (< 0.05), CFI (> 0.95), TLI (> 0.95) (Hu and Bentler, 1999; Kline, 2011).^7^ When the fit of the final model was good, this model was used to test the hypotheses and RQ1.

## Results

Because the fits of the submodels were good (see Appendix B), the submodels were merged into the total hypothesized model, which also fit the data well, χ*^2^/df* = 1.850, *p* = 0.003, RMSEA = 0.041, 90% CI [0.02,0.06], *p* = 0.810, CFI = 0.989, TLI = 0.967. Thus, the hypotheses and RQ1 could be tested with this model.

The first hypothesis predicted a positive relation between news consumption during the pandemic and pandemic news avoidance (H1). However, the analysis yielded an opposite relation (β = –0.437, *p* < 0.001). The more often children consumed news during the pandemic, the less often they reported to avoid pandemic news.

Second, we predicted a positive relation between news consumption during the COVID-19 pandemic and (a) negative emotions and (b) anxiety-related behaviors regarding pandemic news more (H2). Results showed that this was neither the case for children’s negative emotions (β = –0.115, *p* = 0.220) nor for their anxiety-related behaviors (β = 0.020, *p* = 0.767). Thus, contrary to our hypothesis, children’s news consumption during the pandemic did not seem to be related to their (a) negative emotions and (b) anxiety-related behaviors regarding pandemic news.

We also predicted a positive relationship between (a) negative emotions and (b) anxiety-related behaviors regarding pandemic news and pandemic news avoidance (H3). The results showed that this was not the case for negative emotions (β = 0.038, *p* = 0.627). However, anxiety-related behaviors were (close to significant) positively related to pandemic news avoidance (β = 0.201, *p* = 0.058), which is in line with the hypothesis that children who experienced anxiety-related behaviors more often, would also avoid pandemic news more often.

For the mediating role of emotional responses regarding pandemic news in the relation between news consumption and pandemic news avoidance, the total and indirect effects did not show indications of mediation. For the relation between news consumption, (a) negative emotions regarding pandemic news and pandemic news avoidance the total effect appeared to be significant (β = –0.441, *p* < 0.001), but the indirect effect was not significant (β = –0.004, *p* = 0.651). The results showed the same for (b) anxiety-related behaviors regarding pandemic news in this relation, i.e., a significant total effect (β = –0.441, *p* ≤ 0.001) and a non-significant indirect effect (β = –0.004, *p* = 0.769). Therefore, a mediating role could not be established.

**FIGURE 2:**
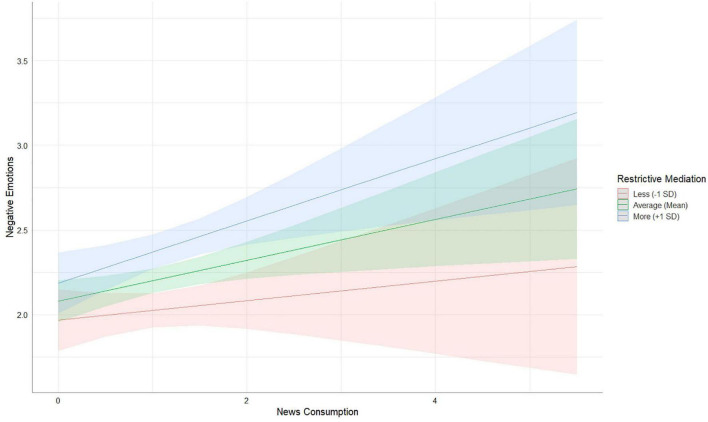
Interaction between news consumption and restrictive mediation on negative emotions. To interpret and plot the interaction effects that emerged from the main analysis, the parental mediation measure was split into 3 levels of restrictive mediation (–1 SD, mean, and +1 SD). This figure was created with package sjPlot (Lüdecke, 2021).

Then, we investigated the moderating role of parental mediation styles for the relation between children’s news consumption and negative emotions regarding pandemic news (H4a). The interaction between news consumption and active parental mediation was not significant (β = 0.016, *p* = 0.472), and neither was the interaction between news consumption and coviewing (β = –0.015, *p* = 0.501). However, the interaction between news consumption and restrictive parental mediation was significant (β = 0.119, *p* < 0.001). As can be seen in the interaction plot in Figure 2, the positive linear relation between children’s news consumption and their negative emotions regarding pandemic news was stronger for children who experienced restrictive mediation relatively more often (+ 1 SD) than children who experienced average or restrictive mediation less often (–1 SD). This supports the hypothesis that children who consumed news more often and reported higher levels of restrictive parental mediation also experienced more negative emotions regarding pandemic news.

**FIGURE 3:**
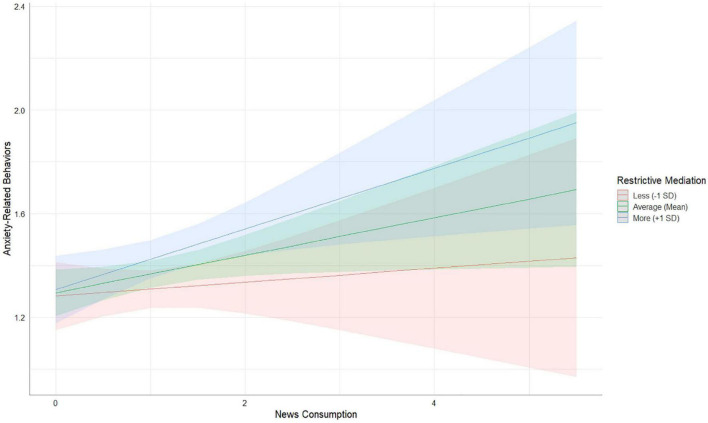
Interaction between news consumption and restrictive mediation on anxiety-related behaviors. To interpret and plot the interaction effects that emerged from the main analysis, the parental mediation measure was split into 3 levels of restrictive mediation (–1 SD, mean, and + 1 SD). This figure was created with package sjPlot (Lüdecke, 2021).

We also investigated the moderating role of parental mediation styles for the relation between children’s news consumption and anxiety-related behaviors regarding pandemic news (H4b). The interaction between news consumption and active parental mediation was not significant (β = –0.011, *p* = 0.505), nor was the interaction between news consumption and coviewing (β = 0.001, *p* = 0.959). Again, the interaction between news consumption and restrictive parental mediation was significant (β = 0.055, *p* = 0.006). Figure 3 shows that the positive linear relation between children’s news consumption and their anxiety-related behaviors regarding pandemic news was stronger for children who experienced restrictive mediation relatively more often (+ 1 SD) than children who experienced average or restrictive mediation less often (–1 SD), supporting the hypothesis that children who consumed news more often and experienced higher levels of restrictive parental mediation also experienced anxiety-related behaviors regarding pandemic news more often.

Regarding the role of reactive coping strategies (H5), we first looked at the direct relation between (a) negative emotions and (b) anxiety-related behaviors and the reactive coping strategies (H5a). Results showed that negative emotions regarding pandemic news were positively related to all four reactive coping strategies, seeking social support in peers (β = 0.300, *p* < 0.001), seeking social support in adults (β = 0.401, *p* < 0.001), distancing (β = 0.363, *p* < 0.001), and externalizing (β = 0.090, *p* = 0.008). In line with the hypothesis, children who experienced more negative emotions regarding pandemic news, were also likely to use reactive coping strategies more often. For anxiety-related behaviors regarding pandemic news this also held for seeking social support in peers (β = 0.310, *p* = 0.003), seeking social support in adults (β = 0.434, *p* < 0.001), and externalizing (β = 0.238, *p* < 0.001), but for distancing it was not significant (β = 0.147, *p* = 0.191).

Moreover, only distancing appeared to have a significant relation with pandemic news avoidance (β = 0.203, *p* < 0.001), whereas seeking social support in peers (β = –0.078, *p* = 0.142), seeking social support in adults (β = –0.050, *p* = 0.296), and externalizing (β = –0.018, *p* = 0.857) did not have a significant relation with pandemic news avoidance (H5b). This indicated that children who used reactive coping strategies more often did not avoid pandemic news less often. To be more specific, distancing even had a significant positive relation with pandemic news avoidance, indicating that children who used distancing more often, also avoided pandemic news more often. In sum, these results do not point toward a decreasing indirect relation, providing no support for H5b.

Looking at the total and indirect relations of the four reactive coping strategies between (a) negative emotions (see Table 2) and (b) anxiety-related behaviors (see Table 3) regarding pandemic news and pandemic news avoidance (RQ1), the results showed that there was only an indirect relation between negative emotions, news avoidance and distancing (β = 0.074, *p* = 0.001). In this relation, there was no role for seeking social support in peers (β = –0.023, *p* = 0.166), seeking social support in adults (β = –0.020, *p* = 0.306), and externalizing (β = –0.002, *p* = 0.858). Moreover, for the relation between anxiety-related behaviors and news avoidance, there was no indirect role for any of the four reactive coping strategies; that is, seeking social support in peers (β = –0.024, *p* = 0.187), seeking social support in adults (β = –0.022, *p* = 0.314), distancing (β = 0.030, *p* = 0.207), and externalizing (β = –0.004, *p* = 0.857). In sum, these results show that only distancing plays a role in mediating the relation between negative emotions regarding pandemic news and pandemic news avoidance.

**TABLE 2:** Total and indirect effects for relations between (a) negative emotions, news avoidance and reactive coping strategies.

	Total effects		Indirect effects	
	β	*p*	β	*p*
Seeking social support in peers	0.014	0.856	–0.023	0.166
Seeking social support in adults	0.017	0.823	–0.020	0.306
Distancing	0.111	0.154	0.074	0.001
Externalizing	0.036	0.639	–0.002	0.858

**TABLE 3:** Total and indirect effects for relations between (b) anxiety-related behaviors, news avoidance and reactive coping strategies.

	Total effects		Indirect effects	
	β	*p*	β	*p*
Seeking social support in peers	0.177	0.098	–0.024	0.187
Seeking social support in adults	0.179	0.090	–0.022	0.314
Distancing	0.231	0.033	0.030	0.207
Externalizing	0.197	0.057	–0.004	0.857

## Discussion

The present study shed light on what makes children avoid the news during a situation with increased impactful and potential frightening news. Although the observed model did not sufficiently explain the mechanisms related to children’s news consumption and news avoidance, our findings can guide future research. Children who consumed more news during the COVID-19 pandemic avoided pandemic news less often. However, children who experienced more anxiety-related behaviors regarding pandemic news avoided pandemic news more often. Dealing with emotional responses elicited by pandemic news could be done by using parent and child mitigation strategies. Restrictive mediation was not an effective parental mediation strategy to temper children’s emotional responses, because the relation between news consumption and emotional responses was stronger for children who experienced restrictive mediation more often. Children with higher levels of emotional responses used reactive coping strategies more often, indicating that they apply these to counteract the negative emotional responses elicited by the news. However, using these coping strategies more often did not seem to be an effective strategy against pandemic news avoidance as none of the coping strategies were negatively related with news avoidance. The only reactive coping strategy that was related to news avoidance – distancing – was even positively related to pandemic news avoidance. To determine the next steps in building a more suitable model, we need to interpret and understand all the observed relations.

### News Consumption and News Avoidance

First and foremost, contrary to our expectations, news consumption during the pandemic related to less instead of more news avoidance. A possible explanation is that news consumption in the current wave is not related to lower levels of news avoidance in the same wave, but possibly in the wave thereafter. This is in line with the study of De Bruin et al. (2021) in which it was found that initial increases of news consumption in the beginning of the pandemic occurred prior to increased news avoidance patterns. So, to allow for cross-lagged modeling we need to include at least two measurements to disentangle the relation and direction between news consumption and news avoidance, preferably three to allow for random-intercept cross-lagged modeling (Hamaker et al., 2015).

### Emotional Responses

Second, more frequent news consumption was not related to more negative emotions and anxiety-related behaviors regarding the pandemic. This might be related to the fact that children in the sample often watch children’s news, which may have played a role in helping them with dealing with the negative elicited by the news. For instance, in children’s news, light and heavy stories are often alternated (the so-called “sandwich formula”; Walma Van Der Molen and De Vries, 2003) and news is often reported in a constructive way in which solutions are provided and positive emotions are included (Kleemans et al., 2017b; Kleemans and Tamboer, 2021). In Dutch children’s news, even special attention is given to how children can deal with unpleasant news (NOS Jeugdjournaal, 2019). It could be that this helps children to deal with the bad things that happen in the world.

Moreover, anxiety-related behaviors were overall very low, which might demonstrate that pandemic news barely affected children in this respect. An explanation may lie in the concept of desensitization, which entails long-term decreased emotional responses to violent (news)media consumption (Scharrer, 2008). As data for this study were collected in an advanced stage of the COVID-19 pandemic (approximately 8 months after the start of the first lockdown), this emotional tolerance might also be developed by children’s repeated exposure to non-violent, but still negative, news media. Further research could explore desensitization to this kind of negative news.

Unlike Skovsgaard and Andersen’s (2020) predictions that negative news would negatively affect news consumers’ well-being, and people would therefore choose to avoid the news, we found no evidence that children who experienced more negative emotions also avoided pandemic news more often. A possible explanation is that experiencing negative emotions on its own does not explain negative news avoidance, but that when children repeatedly experience negative emotions, their well-being is negatively affected (cf. Skovsgaard and Søberg, 2016; Boukes and Vliegenthart, 2017, in Skovsgaard and Andersen, 2020), which on the longer term could lead to news avoidance. It would thus be interesting to investigate the long-term relations between experiencing negative emotions and news avoidance, including subjective well-being as a mediator. In the present study, we did find that children who experienced higher anxiety-related behaviors were more likely to avoid the news. It might be that anxiety-related behaviors are more intense and impactful than experiencing negative emotions. Related to that, we only investigated one of the three predictors that Skovsgaard and Andersen (2020) discerned as being of influence on news avoidance. Future studies should further investigate trust in news and feelings of news overload as well to paint the bigger picture when it comes to (children’s) news avoidance.

### Parent and Child Mitigation Strategies

Regarding the parental mediation strategies, the association between children’s news consumption and negative emotions and anxiety-related behaviors was strongest for children who experienced relatively more restrictive mediation from parents – which is in line with findings from Buijzen et al. (2007) and Morelli et al. (2022). This points toward a boomerang effect of restrictive mediation as discussed in mediation literature before (White et al., 2015; Padilla-Walker et al., 2016). Additionally, a study of Fikkers et al. (2017) showed that these boomerang effects only occur when parents use an inconsistent style for restriction, but when autonomy-supportive restriction is applied it successfully results in reducing negative behaviors. Buijzen et al. (2007) suggested that when children are still exposed to the news – despite the restrictions from parents –, they might not be able to talk about it with their parents, which in turn could maintain their higher emotional responses to the news. Moreover, previous findings on coping with emotions revealed that trying to make negative emotions disappear can backfire, while the acceptance of negative emotions is related to well-being (for a review, see Gruber et al., 2011). Parents who try to prevent their children from experiencing negative emotions might hold their children back from accepting that bad things are happening.

Unlike Buijzen et al. (2007) and Morelli et al. (2022), active mediation did not affect the relation between children’s news consumption and negative emotions or anxiety-related behaviors; nor did coviewing. It might be insightful to explore parents’ experience on practicing parental mediation strategies as well, by providing parent-reports in future research. Moreover, the quality of parents’ explanations or comments to the news differ between families and might influence children’s emotional responses as well. In line with Nathanson and Eveland (2019), the nature of the comments provided by parents – i.e., positive, negative or neutral – deserves more attention in future studies. The same holds for the way parents restrict their children in their news consumption, e.g., inconsistent or autonomy-supportive restrictive mediation (Fikkers et al., 2017). Therefore, qualitative research in the form of interviews or daily diaries could provide the field with relevant insights into this concept.

Finally, children indeed used coping strategies more often when they experienced more negative emotions and anxiety-related behaviors. This adds to knowledge about coping in children, that news can also be seen as a stressful situation and perhaps dealt with by applying reactive coping strategies (Causey and Dubow, 1992). However, none of the reactive coping strategies seemed to be effective in reducing pandemic news avoidance. Only distancing related positively to news avoidance. This adheres to Hoffner and Haefner (1994) who argued that news avoidance might give children the chance to use coping strategies like distraction to prevent them from getting more upset by the news. These findings give rise to the question whether news avoidance might be a form of distancing and could be a coping strategy, rather than an outcome. This is also related to the qualitative study of Ytre-Arne and Moe (2021), in which they argue that in these pandemic times citizens who are very much engaged choose to avoid the news to cope with negative emotions. Therefore, longitudinal data and cross-lagged modeling could provide us with the causal direction of coping and news avoidance, and how successful applying certain coping strategies is in experiencing fewer negative emotions and practicing news avoidance less often over time.

### Limitations and Future Directions

There are several limitations to this study that should be mentioned. First, the relation between news consumption and news avoidance was not measured on an equal level; that is, news consumption was measured in general (albeit during the pandemic), whereas news avoidance was measured specifically for pandemic news. Moreover, although we used the items for intentional news avoidance based in the study of Van den Bulck (2006), the measurement did not seem ideal. To be more concrete, the items seem to reflect reasons to avoid the news – such as “It is not interesting enough or it bothers me” –, focus on occasional news avoidance instead of structural news avoidance, and one item even seems to reflect unintentional news avoidance (“There is so much to follow in the media, that I seldom follow the news about COVID-19”), according to the framework of Skovsgaard and Andersen (2020). For the conceptualization of news avoidance, it might be insightful to investigate the general concept of news avoidance qualitatively, to use as a basis for future quantitative studies regarding this topic.

Additionally, although the pandemic served as a strong context to investigate news-related mechanisms and identify how such a highly impactful (news) event affects children, the question remains whether these results can be generalized to a more “regular” context with several other news events – of which some have less impact on children’s lives –, or events that only need news coverage for a shorter period. For example, it is worth investigating how children react to negative news when the news is not flooded with pandemic news, but with other (negative) news events or how children respond to an acute, major news event. Coping with other (negative) news events might elicit different emotional responses and deserves alternative parent and child mitigation strategies.

Finally, an important limitation concerns the cross-sectional data used for this study. Because all variables were examined at the same time, causal relations cannot be determined. However, these cross-sectional data were of great value, because they allowed to test the basic assumptions in a large sample. We now have a better idea of how these news-related variables are related to one another and which specific mechanisms deserve more research attention. Therefore, this study serves as a promising starting point in research concerning (children’s) news avoidance.

## Conclusion

To conclude, although the current study was not able to fully unravel how news avoidance-related constructs relate to one another, we were able to get some important insights for future research. Especially the relationship between news consumption and news avoidance should be explored more in-depth as these concepts might influence one another over time, instead of at one particular moment (i.e., one specific measurement). In addition, reactive coping strategies seem to be important for further exploration. Still, the role of parents should not be ignored, and we need to dive into the quality and effectiveness of the mediation strategies parents apply to mediate children’s (news) media consumption. All in all, the concept of news avoidance deserves more research attention in general when it comes to the conceptualization and the consequences on the long-term. Thus, the findings of this study call for a longitudinal, cross-lagged analytical approach enriched with qualitative research. Specifically, it is of crucial importance to unravel the mechanisms that increase the chance of children’s news avoidance and those that mitigate it, to build interventions to counteract news avoidance and protect children from the negative emotional consequences by (negative) news consumption.

## Data Availability Statement

The original contributions presented in the study are included in the article/Supplementary Material, further inquiries can be directed to the corresponding author/s.

## Ethics Statement

The studies involving human participants were reviewed and approved by the Ethics Committee of the Faculty of Social Sciences of Radboud University (ECSS). Written informed consent to participate in this study was provided by the participants’ legal guardian/next of kin.

## Author Contributions

ME conceived of the study, participated in the design and coordination, data collection, performed the statistical analysis, and drafted the manuscript. MB and MK conceived of the study, participated in the design of the study, interpretation of the data, and helped to draft the manuscript. RL participated in the design of the study, interpretation of the data, and helped to draft the manuscript. All authors read and approved the final manuscript.

## Conflict of Interest

The authors declare that the research was conducted in the absence of any commercial or financial relationships that could be construed as a potential conflict of interest.

## Publisher’s Note

All claims expressed in this article are solely those of the authors and do not necessarily represent those of their affiliated organizations, or those of the publisher, the editors and the reviewers. Any product that may be evaluated in this article, or claim that may be made by its manufacturer, is not guaranteed or endorsed by the publisher.

## Footnotes

1 When designing this study, concerns regarding children’s news avoidance were mainly based on children’s emotional responses after news exposure (Alon-Tirosh and Lemish, 2014). Therefore, the other two reasons Skovsgaard and Andersen (2020) discerned – people’s trust in news and feeling overloaded by it – were not investigated in the current study.

2 In our preregistration, we used the term “COVID-19” instead of “COVID-19 pandemic”. We changed this in all hypotheses, because “COVID-19” only refers to the disease, whereas “pandemic” involves also issues related to the disease and is thus more suitable here.

3
https://osf.io/ax9cw

4
https://easy.dans.knaw.nl/ui/datasets/id/easy-dataset:238924

5
https://osf.io/d76n2/

6 In the originally preregistered plans, general negative emotions children experienced in the week they filled out the questionnaire would be included as a covariate. However, due to a planning error, this variable was not measured in Wave 2 and, thus, could not be added to the model as covariate (see note in preregistration).

7 In our preregistration we stated that one of the GOF-indices to evaluate the model would be χ *^2^* < 0.05. Given its better suitability for large datasets, we adjusted this to χ *^2^/df* (Hu and Bentler, 1999).

## Appendix A

**APPENDIX TABLE A1:** Zero-order correlations for all study’s variables.

Variable	Zero-order correlations													
	1	2	3	4	5	6	7	8	9	10	11	12	13	14
1. NC	–													
2. NE	0.142**	–												
3. ARB	0.124*	0.480***	–											
4. NAV	-0.251***	0.013	0.066	–										
5. AM	-0.003	0.108*	0.022	-0.042	–									
6. RM	0.101*	0.244***	0.121**	-0.008	0.309***	–								
7. CV	0.164**	0.052	0.047	-0.252***	0.304***	0.160**	–							
8. CSA	0.144**	0.330***	0.301***	-0.044	0.179***	0.282***	0.149**	–						
9. CSP	0.184***	0.310***	0.263***	-0.078	0.063	0.095*	0.119*	0.548***	–					
10. CD	0.054	0.249***	0.175***	0.195***	0.041	0.133**	-0.028	0.305***	0.265***	–				
11. CE	0.050	0.224***	0.303***	0.029	-0.050	0.083	-0.052	0.112*	0.121*	0.127**	–			
12. Sex	0.073	0.253***	0.137**	-0.074	0.020	0.053	0.040	0.044	0.258***	0.082	0.020	–		
13. Grade	0.084	-0.100*	-0.021	-0.019	0.042	-0.115*	0.041*	-0.050	0.069	-0.011	0.034	0.005	–	
14. Prox	-0.018	0.087	-0.011	-0.040	0.094*	0.062	0.067	-0.026	0.034	-0.035	-0.060	0.071	-0.068	–

*Pearson’s r correlations for all variables using the total sample (n = 510).*

*NC: News consumption, NE: Negative Emotions, ARB: Anxiety-Related Behaviors, NAV: News Avoidance, AM: Active Mediation, RM: Restrictive mediation, CV: Coviewing, CSA: Coping Seeking Social Support of Adults, CSP: Coping Seeking Social Support of Peers, CD: Coping Distancing, CE: Coping Externalizing, Prox: Proximity of COVID-19.*

**p < 0.05. **p < 0.01. ***p < 0.001 (two-sided).*

## Appendix B

As explained in the analysis procedure, we first tested submodels prior to testing the full model. We tested three submodels, one for news consumption, news avoidance and (a) negative emotions and (b) anxiety-related behaviors as mediators (submodel 1), the second one for parental mediation as moderator for the relation between news consumption and (a) negative emotions and (b) anxiety-related behaviors (submodel 2), and the third model for the mediating relation of reactive coping strategies in the relation between (a) negative emotions, (b) anxiety-related behaviors and news avoidance (submodel 3). These submodels were also tested using Structural Equation Modeling in R (Lavaan package; Rosseel, 2012). Full information maximum likelihood (FIML) was used as the imputation strategy and a robust estimator (MLR) was used. For assessing whether the model adequately fit the observed data, we looked at Goodness-of-Fit indices, which were χ*^2^*/df < 3, RMSEA (< 0.05), CFI (> 0.95), TLI (> 0.95) (Hu and Bentler, 1999; Kline, 2011). If the fit of the (sub)model was not adequate, we looked at the modification indices (MI > 3.84; Lei and Wu, 2007) to see which paths should be added to the model to improve the fit.

The first submodel, adequately fit the data according to three out of four criteria, χ *^2^/df* = 1.516, *p* = 0.208, RMSEA = 0.032, 90% CI [0.00,0.09], *p* = 0.631, CFI = 0.993, TLI = 0.950 (TLI should be > 0.95). The second submodel, fit the data well, χ *^2^/df* = 1.275, *p* = 0.225, RMSEA = 0.023, 90% CI [0.00,0.05], *p* = 0.921, CFI = 0.998, TLI = 0.995. The third submodel, also showed a good fit with the data, χ *^2^/df* = 1.558, *p* = 0.197, RMSEA = 0.033, 90% CI [0.00,0.09], *p* = 0.618, CFI = 0.997, TLI = 0.957.
