# Supplementary material for: Explaining Children’s News Avoidance During the COVID-19 Pandemic
Source: Front Psychol. 2022 Jun 22;13:889096. doi: 10.3389/fpsyg.2022.889096 (PMC9258973; doi:10.3389/fpsyg.2022.889096)
Supplement: Supplementary file 1 [file Data_Sheet_1.pdf]

Supplementary Material

Figure 1
Conceptual Model

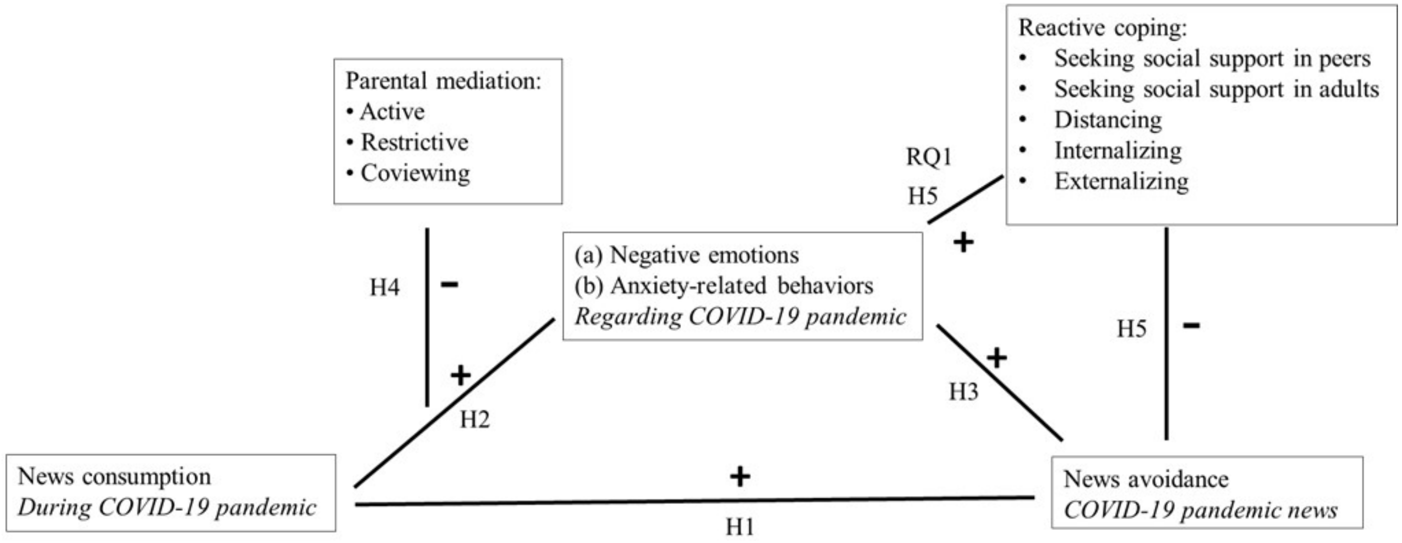

Figure 2

Interaction between News Consumption and Restrictive Mediation on Negative Emotions.

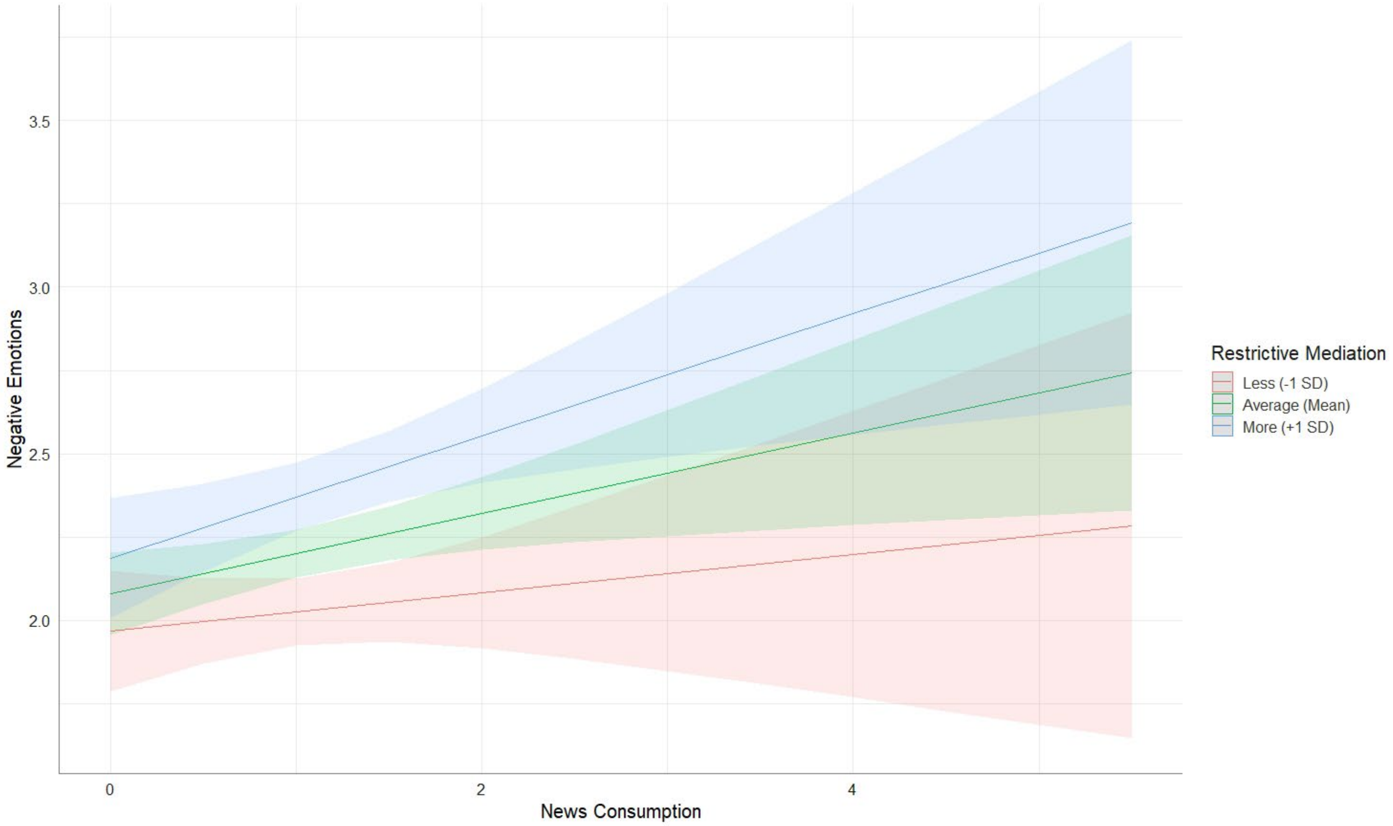

Note. To interpret and plot the interaction effects that emerged from the main analysis, the parental mediation measure was split into 3 levels of restrictive mediation (-1 SD, mean, and +1 SD). This figure was created with package sjPlot (Lüdecke, 2021).

Figure 3

Interaction between News Consumption and Restrictive Mediation on Anxiety-Related Behaviors.

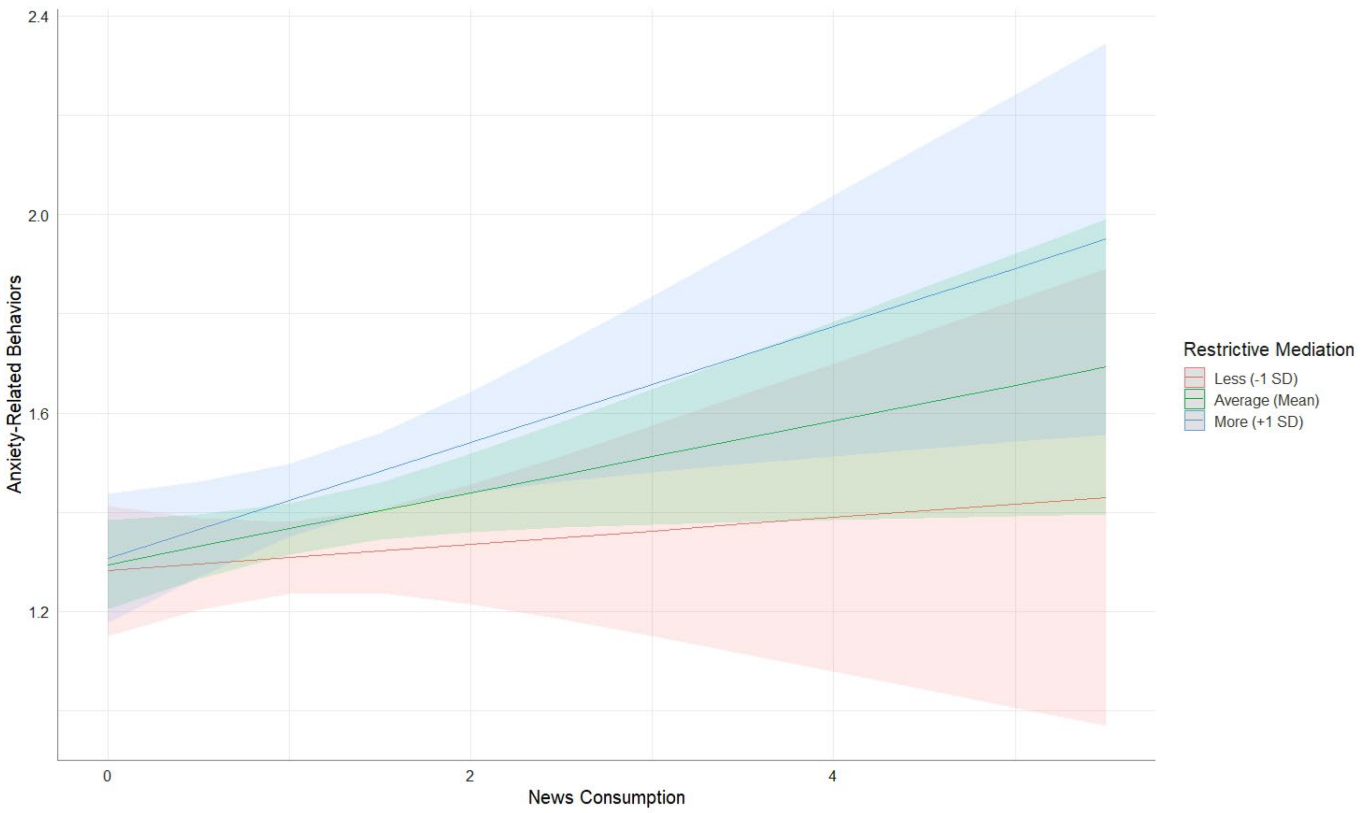

Note. To interpret and plot the interaction effects that emerged from the main analysis, the parental mediation measure was split into 3 levels of restrictive mediation (-1 SD, mean, and +1 SD). This figure was created with package sjPlot (Lüdecke, 2021).
